# Severe drought impacts tree traits and associated soil microbial communities of clonal oaks

**DOI:** 10.1186/s40793-025-00720-7

**Published:** 2025-06-06

**Authors:** Camilo Quiroga-González, Luis Daniel Prada-Salcedo, François Buscot, Mika Tarkka, Sylvie Herrmann, Marie-Lara Bouffaud, Kezia Goldmann

**Affiliations:** 1 Department of Soil Ecology, UFZ - Helmholtz Centre for Environmental Research, Theodor-Lieser-Straße 4, 06120 Halle (Saale), Germany; 2 German Centre for Integrative Biodiversity Research (iDiv) Halle-Jena-Leipzig, Puschstraße 4, 04103 Leipzig, Germany

**Keywords:** *Quercus robur*, Soil bacteria, Soil fungi, Plant traits, Environmental stress

## Abstract

**Background:**

Biotic and abiotic factors, including plant age, soil pH, soil organic matter concentration, and especially water availability, significantly influence soil microbial populations and plant characteristics. While many ecosystems are adapted to occasional droughts, climate change is increasing the frequency and severity of drought events, which negatively impacts plant productivity and survival. Long-lived, drought-sensitive tree species such as *Quercus robur* are particularly vulnerable to water shortages. Drought also alters soil microbial communities, reducing and reshaping microbial diversity, biomass, and activity, which can in turn disrupt key ecosystem functions. The objective of this study was to investigate the effects of natural drought conditions on soil physicochemical variables, plant traits and microbial communities of the oak clone DF159 in Central Germany. Our research focuses on two study sites, Bad Lauchstädt and Kreinitz, which differ in soil water retention capacity. Data collection spans two periods: before and after a severe drought in 2018. Oak traits and environmental data was collected from 2011 to 2023 covering two oak time series with trees planted annually between 2010 and 2019. Microbial communities were analyzed every second year between 2015 and 2021 around trees representing five different ages.

**Results:**

We found that plant traits, including apical growth, branch elongation and number of shoot flushes, were positively correlated with precipitation and relative humidity. Although the study sites differed in oak leaf number per shoot flush and number of shoot flushes, the 2018 drought negatively impacted all measured plant traits, regardless of sites. Soil bacterial richness and diversity declined at both study sites, independent of plant age, while fungal richness specifically increased in Bad Lauchstädt, which has a higher water-holding capacity, following the drought event. Bacterial community composition was more strongly affected by drought than fungal communities, whereas the latter was more responsive to plant age than bacterial communities.

**Conclusions:**

Given their strong functional links during drought, interactions among vegetation, microbial communities, and soil functioning may ultimately influence major ecosystem services. Bacterial communities were particularly sensitive to drought, while fungal communities exhibited greater resistance, suggesting their potential role in supporting plant survival under drought stress. These findings highlight the risk that prolonged drought may cause irreversible shifts in microbial communities, with significant implications for soil functions and plant–microbe interactions.

**Supplementary Information:**

The online version contains supplementary material available at 10.1186/s40793-025-00720-7.

## Background

The composition and functionality of soil microbial communities are strongly influenced by both biotic and abiotic factors, particularly vegetation, plant age, soil pH, organic matter content, and most notably, water availability [1, 2]. While most ecosystems are adapted to occasional droughts, the increasing frequency and intensity of drought events due to climate change pose significant challenges for plants [3]. Water limitation can alter vegetation types by affecting plant productivity and survival [4, 5], and warmer temperatures further intensify drought effects [6–10]. Despite their ability to develop extensive root systems that access deeper soil layers, tree communities have shown pronounced vulnerabilities to recent droughts and water deficits, experiencing declines in growth and increased mortality [11–15].

Drought is also associated with shifts in soil microbial communities [16], driven by changes in plant community composition and rhizodeposition [17], as well as by abiotic constraints such as reduced nutrient diffusion [18]. Both, natural and experimental droughts have been shown to decrease soil microbial diversity, biomass and activity [19–23], leading to a significant reduction of key ecosystem functions such as carbon cycling, nitrogen mineralization and phosphorus solubilization [19, 24–27]. Consequently, soils under drought conditions often exhibit lower nutrient availability [19, 25, 28, 29].

Microorganisms employ various strategies to overcome or resist drought: Some form spores that remain dormant during water deficits [30–32] and germinate after soil rewetting [33–35]. Gram-positive bacteria are generally more drought-resistant due to their thicker cell walls [18]. Similarly, certain fungi are less affected or even favored by drought compared to prokaryotes [32, 36–40]**.** Fungal adaptations such as thicker cell walls, melanin, and osmolytes help maintain osmotic balance [41], while the hyphal network of filamentous fungi enables access to limited resources under extreme conditions [42], enhancing also their drought resistance.

In 2018, Germany experienced one of its most severe droughts on record, with temperatures exceeding the average by more than 3.3 °C [43]. During this period, many ecologically important tree species showed signs of severe drought stress [15, 43, 44], and soil-borne or root-associated microorganisms were also affected [15, 45]. However, currently little is known about how the presence of trees alter the soil microbial communities during this specific drought event.

Accordingly, this study aims to elucidate the effects of the 2018 drought on soil physicochemical properties, tree traits and soil microbial communities. We leveraged two plantations of a clonal oak (*Quercus robu*r L.) used as phytometer, thereby minimizing confounding effects related to plant genotype [46, 47]. The two plantation sites, located in Bad Lauchstädt (Chernozem soil) and Kreinitz (sandy soil) in Central Germany — the region with the country’s lowest precipitation levels — offer contrasting water retention potentials [48, 49]. The Chernozem soil at Bad Lauchstädt retains more water than the sandy soil at Kreinitz, providing a clear contrast in soil water-holding capacities. Given that *Q. robur* is a drought-sensitive tree [50], we anticipated pronounced responses in both the trees and their soil microbiomes to the severe drought event of 2018. We hypothesized that 1) drought-induced changes in soil physicochemical properties and reduced water availability would limit oak growth from 2018 onward, and 2) changes in microbial communities would be mainly associated with plantation site and drought, with the latter having a more negative impact on bacterial diversity than on fungal diversity.

## Methods

### Experimental design

The DF159 clonal oak saplings (*Q. robur*) were produced via micropropagation, which guarantees their common genetic identity [46, 51]. The trees were cultivated in the laboratory and greenhouse before being outplanted at the age of one year. Each year from 2010 to 2019, four oak trees were planted in three separate blocks at two study sites in Central Germany — Bad Lauchstädt in Saxony-Anhalt (51° 23′ 29.65" N, 11° 52′ 32.14" E) and Kreinitz in Saxony (51° 23′ 08.3" N, 13° 15′ 39.7" E). This resulted in twelve trees planted per site per year, using a randomized design in two subplots per site. The first subplot at both study sites consisted of oaks planted annually between 2010 and 2014, while the second subplot included those trees planted between 2015 and 2019. The distance between each tree was set to 50 cm.

### Soil sampling and assessments of oak morphological data

Soil sampling campaigns were conducted in September 2015, 2017, 2019 and 2021, targeting a subset of trees that were between one to five years old at the time of sampling.

Soil samples were collected following the approach of Habiyaremye et al*.*, (2020) [47]. From each of the study sites, soil samples from oak individuals were collected within 30 cm distance to the tree trunk at a 0–15 cm depth with an auger. Samples were collected based on the tree age and their respective plot on each site (Table S1). For each tree, three subsamples were taken and subsequently pooled to create composite samples. The composite samples were sieved through a 2 mm mesh to remove roots and organic debris; remaining non-soil particles were manually removed after sieving. The homogenized soil was then divided into two aliquots: one was stored at − 20 °C for molecular analyses, and the other at 4 °C for physicochemical analyses.

Each September from 2011 to 2023, tree performance was assessed to evaluate the effect of environmental conditions. Measurements included total apical growth of the main trunk, trunk diameter at 150 mm and 1300 mm (DBH) above ground, and the seasonal increase in length of four lateral branches. For each of these branches, shoot flushes produced during the growing season were counted, their respective lengths measured, and the number of leaves recorded. From each shoot flush, five leaves were harvested to determine the mean leaf area as well as mean fresh and dry weights. Besides, we recorded whether tree individuals experienced intraspecific competition due to shading by previously planted trees.

### Determination of soil physicochemical parameters

Soil physicochemical parameters were assessed following Habiyaremye et al*.*, (2021) [52] Soil pH was measured using a glass electrode in a 1:2.5 soil-to-0.01 M CaCl_2_ suspension after one hour of equilibration. Gravimetric soil moisture was assessed with a fully automated moisture analyzer (DBS60-3, KERN & SOHN GmbH, Balingen, Germany), here defined as soil moisture (MOI). Total nitrogen (TN) and total organic carbon (TOC) contents were analyzed in triplicate through dry combustion using a Vario elemental analyzer (EL III, Elementar, Hanau, Germany), and the carbon-to-nitrogen ratio (TOC/TN) was subsequently calculated from these values. To evaluate the potentially bioavailable fraction of soil organic carbon and nitrogen for microbial activity, hot water-extractable carbon and nitrogen (HWC and HWN, respectively) were measured according to Ghani et al*.* (2003) [53] and Schulz et al*.* (2011)[54]. Additionally, labile organic carbon and nitrogen, i.e. easily decomposable by soil microorganisms, were measured as cold water-extractable carbon (CWC) and nitrogen (CWN) following the previously described procedures [55–57]. Following Habiyaremye et al*.* (2021) [52], ammonium (NH_4_^+^-N) and nitrate (NO_3_^−^ − N) were quantified, and their sum represents the total mineral nitrogen content (N_min_).

### Molecular sample processing and amplicon sequencing

The DNA extraction and sequencing preparation followed the descriptions in Habiyaremye et al*.*, (2020) [47]**.** In brief, DNA was extracted from 0.4 g of soil sample using the Power Soil DNA Isolation Kit (Qiagen, Hilden, Germany), with extracts being quantified using a NanoDrop spectrophotometer. DNA was then adjusted to 10–15 ng/μl. For assessing bacterial communities, the V4 region of the 16S rRNA gene using 515F/806R primers was amplified [58, 59]. The primer pair ITS7/ITS4 [60–62] was used to amplify the internal transcribed spacer (ITS) region 2 to access fungal communities. Amplifications followed the conditions described in Habiyaremye et al*.*, (2020) [47], with three replicates per sample. PCR products were barcoded and pooled in equimolar concentrations, purified, and indexed using Nextera XT adaptors. After a second purification, DNA libraries were quantified, pooled to equal concentrations, and assessed for fragment size and quality. Finally, libraries were sequenced using a 2 × 300 bp paired-end MiSeq Reagent kit on an Illumina MiSeq platform at the Soil Ecology Department of the UFZ—Helmholtz Centre for Environmental Research in Halle (Saale), Germany.

### Bioinformatics

Raw sequencing data was processed using the dadasnake pipeline (v0.11.2, [63]) with default settings. This pipeline is based on the workflow built for the DADA2 algorithm [64]. The processing included several steps: quality filtering, where reads were truncated based on quality scores of 13 for 16S and 15 for ITS and sequences below these thresholds were removed; error model learning, in which DADA2 modeled and corrected sequencing errors; and dereplication, where identical reads were collapsed into amplicon sequence variants (ASV). ASV were clustered into operational taxonomic units (OTU) considering a sequence similarity of 97% [65, 66]. Chimeric sequences were identified and removed using the DADA2 consensus method. Taxonomic classification was then performed by assigning sequences to taxa using SILVA (v 138.1; [67]) for 16S sequences, and UNITE (v 10.0; [68]) for ITS sequences.

### Data processing of environmental parameters

Environmental data from 2010 to 2023 was analyses to assess changes in precipitation and temperature across two study sites, Bad Lauchstädt and Kreinitz. Data was filtered annually including only the months from May to August, corresponding to the main tree growth period. To facilitate the analyses, cumulative values were calculated for total precipitation, while mean values were determined for relative humidity, temperature, maximum temperature and minimum temperature.

To examine changes in environmental variables over time and between sites, an analysis of variance (ANOVA; v 4.3.1; [69]) was conducted. Years were then grouped into one categorical variable called *drought period* with two levels: before the drought (pre 2018) and after the drought (post 2018). A separate linear mixed model (lme4 package v. 1.35.1; [70]) was used to determine the effect of study sites and drought period on soil physicochemical variables, using them as fixed effects. Tree age, light competition (shading by adjacent trees), and plot were included as random effects. For the drought mixed model, study site was also included as a random effect. Soil physicochemical variables that were not normally distributed were log-transformed prior analyzing.

### Data processing of plant traits

For the analyses of DF159 plant traits, data collected from 2010 to 2023 was used. To address collinearity, plant traits exhibiting correlations greater than 80% with any other trait were excluded, resulting in a final set of seven plant traits for evaluation: leaf area, number of leaves per shoot flush, leaves dry weight, number of shoot flushes, branch elongation, apical growth, and the average length of the shoot flushes. To determine differences between the study sites and the potential effect of the drought on these plant traits, we performed linear mixed models, allowing for a comprehensive assessment of how drought and site impact each trait. Partial correlation analysis [71], accounting for study site, were conducted between environmental variables and plant traits. This approach enabled us to isolate the direct association between oak traits, while controlling for potential confounding effects introduced due to sites differences. A heatmap was generated to visualize variables that showed significant statistical correlations (*p-*value < 0.05).

### Data processing of microbial sequencing data

The bacterial and fungal OTU tables were exported to R (v 4.3.1; [69]) and processed with the phyloseq package (v 1.46.0; [72]). 16S and ITS data was rarefied to 37,714 and 5,192 sequences per sample, respectively. OTUs without phylum assignment, and bacterial OTUs classified as chloroplasts were removed, resulting in 226 samples (114 samples from Kreinitz and 112 from Bad Lauchstädt) for subsequent analyses. Alpha diversity was assessed using Hill numbers [73] via the package hilldiv (v 1.5.1; [74]). To determine the effect of drought on microbial richness and diversity, a linear mixed model (lme4 package v.1.35.1; [70]) was performed using drought as fixed effect and age, site, competition and plot as random effects. Linear models tested for independent associations between Hill numbers and categorical variables (study site, year of sampling, year of out-planting, plot, competition and age of the trees). Beta diversity analysis was based on Bray–Curtis distances. Non-metric multidimensional scaling (NMDS) was used to visualize microbial community patterns, while variance partitioning statistically tested the effects of the categorical variables on microbial community composition while addressing potential collinearity. Rank abundance curves (BiodiversityR package v 2.15.4; [75]) determined shifts in dominant orders across the categorical variables. A canonical analysis of principal coordinates (CAP; ordinate function from the phyloseq package) was performed to explore the relationship between environmental variables and microbial communities. Differential abundance analysis using linear discriminant analysis effect size (LefSe) [76] identified microbial orders significantly differing between drought periods. Microorganisms, which were significantly affected (*p* ≤ 0.01) were visualized in a heatmap and correlated with soil physicochemical variables. To ensure robustness, only microorganisms that show consistent correlation trends across study sites and drought periods were retained for interpretation.

## Results

This study evaluated how environmental and soil physicochemical variables influence the oak clone DF159, planted as a phytometer in pure stands, by examining plant growth, plant traits, and associated soil bacterial and fungal communities. We compared two study sites in Central Germany with contrasting soil water retention potentials across two time periods: 2015 to 2017 and 2019 to 2021, separated by a severe drought event in 2018. Climatic data, i.e. total precipitation and relative humidity, confirmed the significant impact of the drought in 2018 at both study sites (Figure S1).

### Study site and drought effects on soil physicochemical properties

Linear mixed models revealed significant differences in soil properties between study sites: Bad Lauchstädt showed higher values for pH, MOI, NO_3_^−^ − N, CWC/CWN, TOC, TN and TOC/N compared to Kreinitz (Table S2). In contrast, Kreinitz had higher values of DMC, NH_4_^+^-N, HWC and HWC/HWN than Bad Lauchstädt. The models also indicated differences in soil chemical properties before and after the drought, with higher values of MOI, NH_4_^+^-N, NO_3_^−^ − N, N_min_ and TOC/TN before the drought, while CWC, CWC/CWN and TN were higher after the drought (Table S3).

### Study site and drought effects on oak traits

**Fig. 1:**
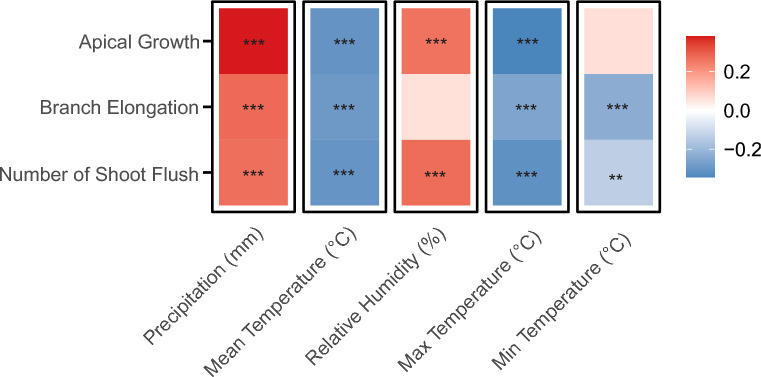
Correlations between climatic variables and plant traits. Red colors represent a positive correlation, while blue represents a negative one. Asterisks indicate significance level, i.e. *p-*value: *0.01 **0.001 ***0.0001

Oaks in Bad Lauchstädt produced significantly more leaves per shoot flush and a higher number of shoot flushes compared to those in Kreinitz (Table S4). Across both study sites, all measured plant traits were influenced by the drought period (pre and post 2018; Table S5). Most plant trait values were higher before the drought, except for leaf area, which increased after the drought. Partial correlation analyses showed that the number of shoot flushes, apical growth, and branch elongation were positively correlated with precipitation (Fig. 1). The number of shoot flushes and annual apical growth were also positively correlated with relative humidity. In contrast, these three traits showed negative correlations with mean and maximum daily temperature, while minimum daily temperature was negatively correlated with number of shoot flushes and branch elongation.

### Study site and drought effects on soil microbial diversity

**Fig. 2:**
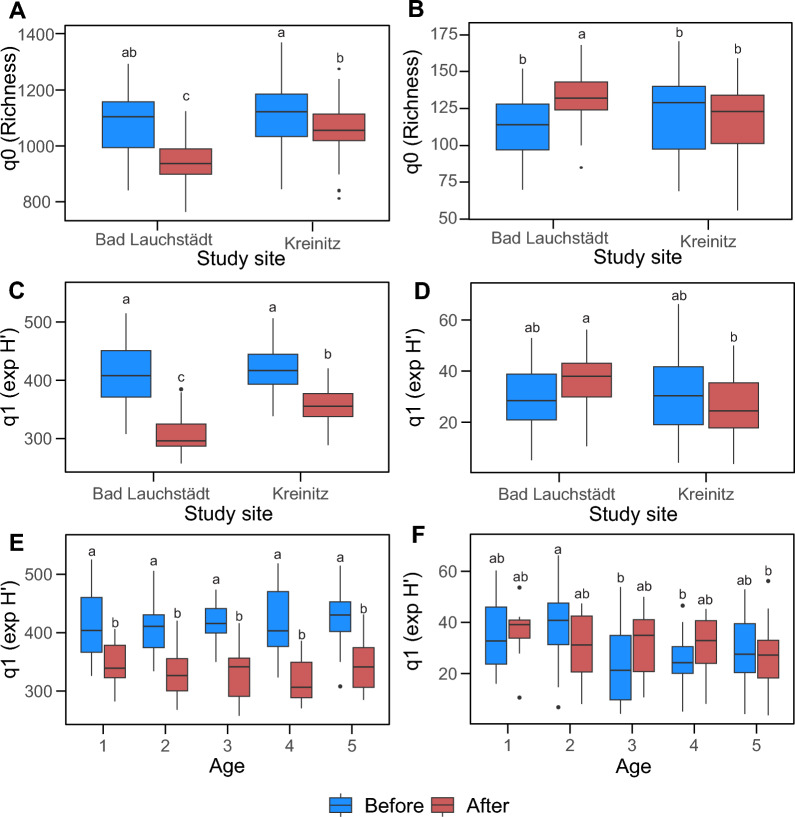
Boxplots showing the distribution of richness (q0 according to Hill numbers) and exponential Shannon diversity (q1 according to Hill numbers) before and after the drought in 2018. **A** bacterial richness for both study sites, **B** fungal richness for both study sites, **C** bacterial Shannon index for both study sites, **D** fungal Shannon index for both study sites, **E** bacterial Shannon index at each oak age, and **F** fungal Shannon index at each oak age. Different letters above boxplots indicate significant differences between treatments tested

Bacterial richness was significantly higher in Kreinitz compared to Bad Lauchstädt (F = 27.66, *p* < 0.01). Linear mixed models revealed significant differences in bacterial richness across drought periods, with a higher bacterial richness observed before the 2018 drought (*p* < 0.01, R^2^ = 0.49, t = -6.07) (Fig. 2A). We also found a significant interaction between study sites and drought period, indicating that Bad Lauchstädt was more affected by the drought than Kreinitz (F = 11.99, *p* < 0.01) (Fig. 2A). Year-by-year analyses confirmed these patterns: bacterial richness was higher in 2015 and 2017 (pre-drought) than in 2019 and 2021 (post-drought).

For fungi, linear mixed models showed no significant differences in richness due to drought (*p* = 0.49, R^2^ = 0.02, t = 2.70) (Fig. 2B) or between study sites (F = 2.02, *p* = 0.156). However, when analyzed separately by site, fungal richness in Bad Lauchstädt increased significantly after the drought (Fig. 2B; F = 12.53, *p* < 0.01).

Analysis of the exponential Shannon indices for bacteria and fungi showed similar trends to those observed for richness. For bacteria, drought had a significant negative effect on Shannon diversity (*p* < 0.01, R^2^ = 0.58, t = − 15.24) (Fig. 2C). We also found a significant interaction between drought and study site (Fig. 2C; F = 38.94, *p* < 0.01), and between drought and tree age (Fig. 2E; F = 2.50, *p* = 0.04). Bacterial Shannon diversity was constantly higher before the 2018 drought across both sites and all tree ages. In contrast, fungal exponential Shannon indices did not differ before and after the drought (Fig. 2D; *p* = 0.31, R^2^ = 0.003, t = 0.99), and no interactions with study site or tree age were observed (Fig. 2D, F).

For the inverse Simpson index, bacterial diversity was higher before the 2018 drought (*p* < 0.01, R^2^ = 0.25, t = − 4.27). Kreinitz showed marginally higher bacterial diversity compared to Bad Lauchstädt (*p* = 0.051, R^2^ = 0.012 F = 3.85) (Figure S2A), with no difference between the tree age (Figure S2B; *p* = 0.377, R^2^ = 0.001, F = 1.06). Regardless of study site, drought negatively affected bacterial diversity (Figure S2A). For fungi, no significant differences in diversity were found between drought stages (*p* = 0.44, R^2^ = 0.0019, t = 0.77).

### Study site and drought effects on soil microbial community composition

The bacterial and fungal community compositions differed significantly between study sites, as shown by the NMDS analysis (Figure S3). Variance partitioning analysis indicated that site (*p* < 0.01, R^2^ = 0.79) and drought (*p* < 0.01, R^2^ = 0.014) accounted for the largest proportion of variation in bacterial community composition. For fungal communities, the main explanatory factors were study site (*p* < 0.01, R^2^ = 0.68) and tree age (*p* < 0.01, R^2^ = 0.02). These patterns persisted when analyzing the years separately; 2015 and 2017 differed from 2019 and 2021, but not within those groupings (Figure S4).

**Fig. 3:**
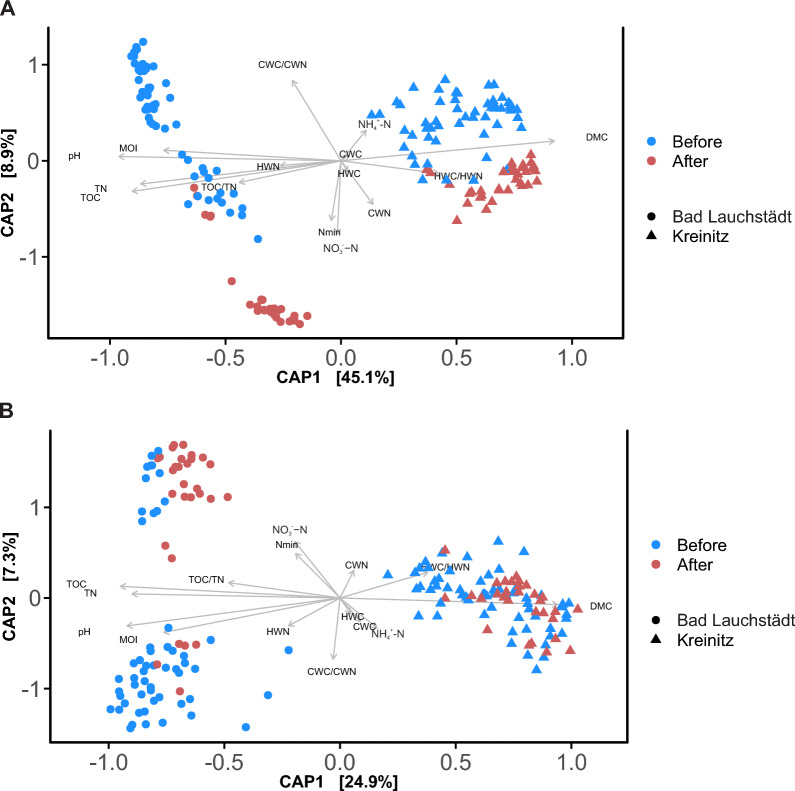
Canonical analysis of principal coordinates (CAP) of the microbial communities based on OTU abundance. The length of each vector indicates the strength of the association between soil physicochemical variable and the community composition of **A** bacteria and **B** fungi

CAP analysis further revealed differences in bacterial communities associated with the study site along axis 1 (explaining 45.1% of the variability) and with drought period along axis 2 (explaining 8.9% if the variability) (Fig. 3A). The length of the vectors indicated that pH, TN, TOC, MOI and DMC were most strongly associated with differences between study sites, while NO_3_^−^ − N, N_min_ and CWC/CWN were more associated with differences between drought periods. For fungi, similar results were found, axis 1 (explaining 24.9% of the variation) was more strongly associated to study site, while axis 2 explained 7.3% of the variation (Fig. 3B). For fungal communities, DMC, TOC, TN, pH and MOI best explained study site differences, while none of the measured variables stood out between drought periods.

**Fig. 4:**
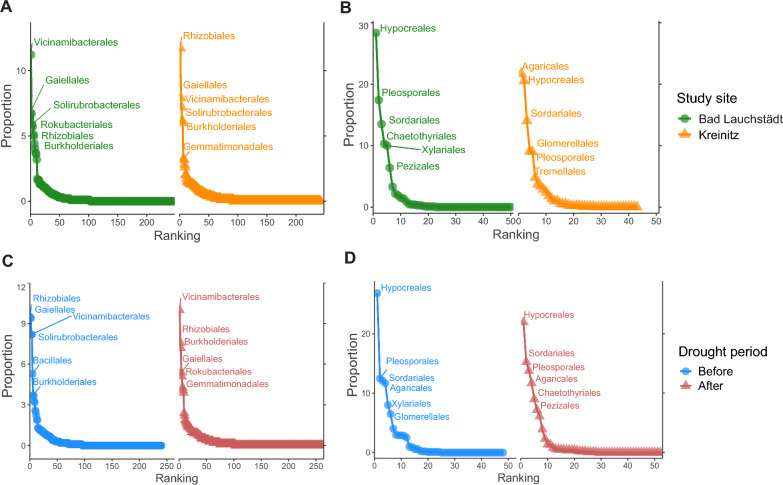
Rank abundance curve based on microbial order. The curves show the six most abundant bacterial orders for **A** each study site, and **B** each drought period, and the six most abundant fungal orders for **C** each study site and **D** each drought period

Rank abundance curves at order level showed differences between study sites for bacteria. Bad Lauchstädt harbored 247 different orders and Kreinitz 242 orders, respectively. Most bacterial orders with a frequency above 5% were present in both sites (e.g., Vicinamibacterales, Gaiellales, Solirubrobacterales, Rhizobiales and Burkholderiales), but their relative abundance varied (Fig. 4A). Interestingly, Rokubacteriales represented 5.7% of taxa in Bad Lauchstädt but was not highly abundant in Kreinitz. The abundance of certain orders also shifted after the 2018 drought: Solirubrobacterales, for example, was highly abundant before the drought (8.22%), but declined afterwards. Similarly, Bacillales dropped from the fifth most abundant order before the drought (5.3%) to the thirteenth after the drought (1.7%) (Fig. 4C).

For fungal orders, rank abundance curves showed greater differences between study sites than between drought periods (Fig. 4B, D). In Bad Lauchstädt, Hypocreales dominated (28.3%), followed by Pleosporales (17.4%) and Sordariales (13.5%). In Kreinitz, the dominant orders were Agaricales (21.7%), Hypocreales (20.5%) and Sordariales (12.5%).

**Fig. 5:**
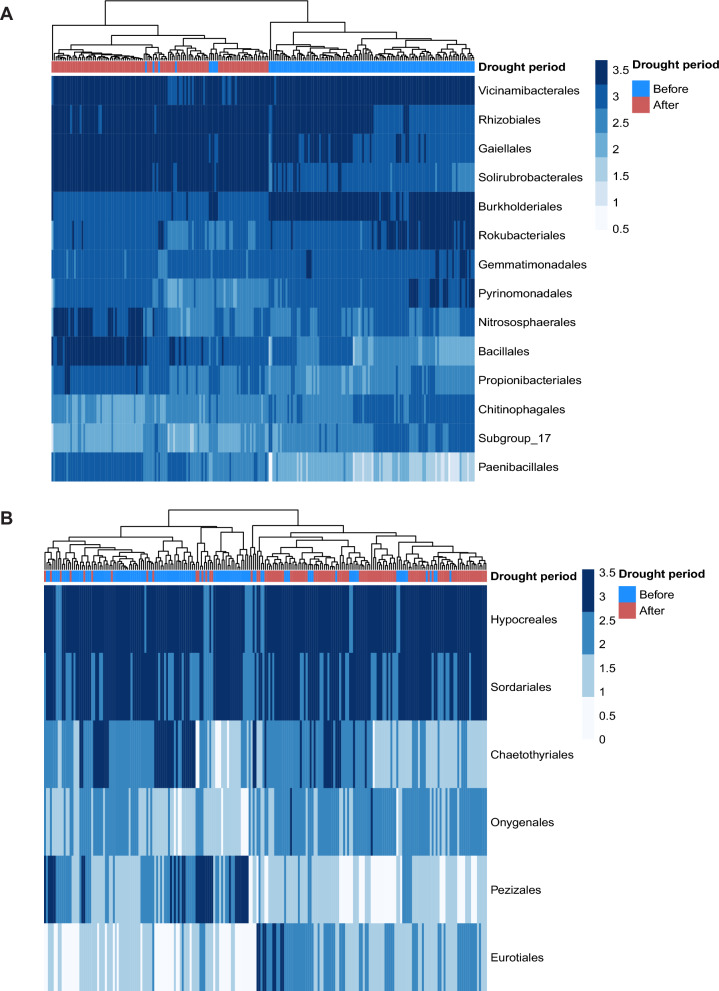
Heatmap showing the **A** bacteria and **B** fungi that are differentially abundant between drought periods. Each cell represents one sample, and the plots are clustered based on their similarity. The blue scale represents the log_10_ abundance of each order

Differential abundances analyses at the order level (*p* < 0.01), determinded by LefSe (Fig. 5), revealed twelve bacterial orders that were significantly affected by the drought. At both sites, Burkholderiales, Gemmatimonadales, Pyrinomonadales, Chitinophagales and Pedosphaerales were more abundant before the drought, while Rhizobiales, Gaiellales, Solirubrobacterales, Nitrosophaerales, Bacillales, Propionibacteriales and Paenibacillales were enriched after the drought (Fig. 5A). For fungi, Sordariales, Chaetothyriales, Pleosporales, Magnaporthales and Pezizales were more abundant before the drought, whereas Eurotiales and Onygenales increased in abundance after it (Fig. 5B).

### Effect of soil physicochemical properties on microbial taxa abundances

**Fig. 6:**
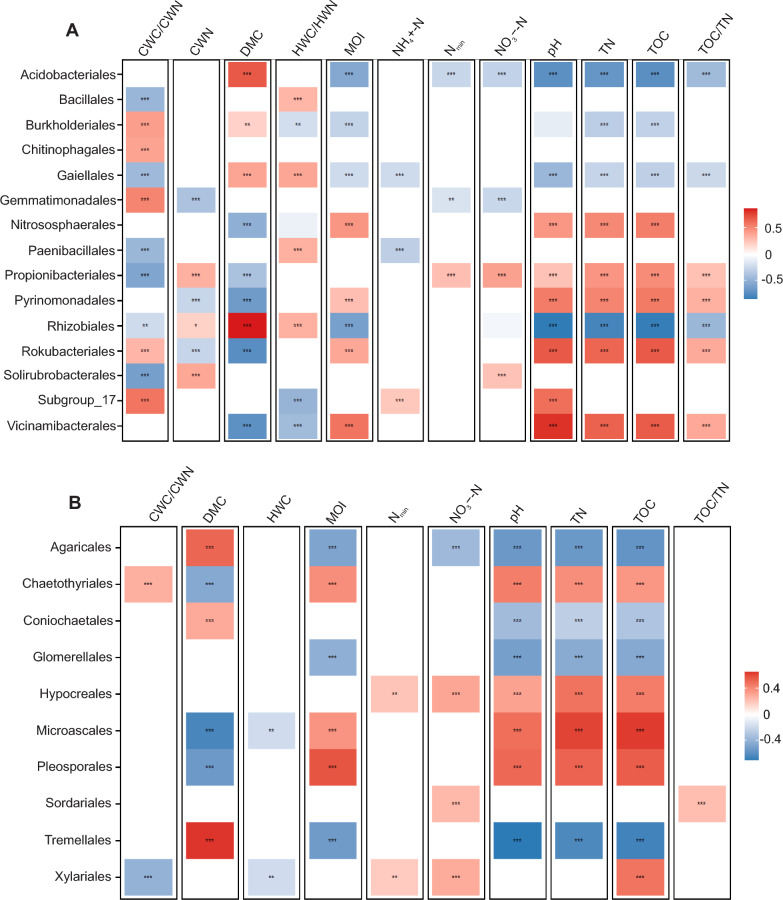
Correlations between soil physicochemical variables and orders of **A** bacteria and **B** fungi. Rows represent the orders that were differentially abundant between study site and drought period. Values correspond to coefficient correlation based on Pearson correlation. Asterisks indicate significance level. *0.01 **0.001 ***0.0001. The abbreviations are associated with the following parameters: Soil Moisture (MOI), Soil Dry Matter (DMC), Soil Dry Matter (DMC), Ammonium Nitrogen Content (NH_4_^+^-N), Nitrate Nitrogen Content (NO_3_^−^-N), Inorganic Nitrogen Content (Nmin), Hot Water Extractable Organic Carbon (HWC), Hot Water Extractable Nitrogen (HWN), Cold Water Extractable Organic Carbon (CWC), Cold Water Extractable Nitrogen (CWN), Total Soil Organic Carbon (TOC) and Total Soil Nitrogen (TN)

Independent of study site and drought period, 15 bacterial orders and ten fungal orders showed significant correlations (*p* < 0.01) with soil physicochemical variables (Fig. 6A–B). Soil pH, TN and TOC were positively correlated with the abundance of Vicinamibacterales (*r* = 0.82, *r* = 0.66 and *r* = 0.68, respectively) and Rokubacteriales (*r* = 0.69, *r* = 0.65 and *r* = 0.68, respectively), and negatively with Rhizobiales (*r* = − 0.86, *r* = − 0.80 and *r* = − 0.84, respectively) and Acidobacteriales (*r* = − 0.72, *r* = − 0.68 and *r* = − 0.73, respectively). In contrast, abundances of Rhizobiales (*r* = 0.87) and Acidobacteriales (*r* = 0.69) displayed positive relationships with DMC, while those of Vicinamibacterales (*r* = − 0.71) and Rokubacteriales (*r* = − 0.74) were negative.

For fungi, only two orders (Tremellales and Microascales) had correlation coefficients higher than 0.6 (Fig. 6B), with positive correlation of DMC and abundance of Tremellales (*r* = 0.66), but negative correlation for Microascales (*r* = − 0.64). On the contrary, TN and TOC showed a positive relationship with Microascales (*r* = 0.62 and *r* = 0.64, respectively), and negative correlation with Tremellales (*r* = − 0.63 and *r* = − 0.66 respectively). Soil pH was negatively correlated with Tremellales (*r* = − 0.70).

Specifically, regarding MOI, abundance of bacterial and fungal orders varied. Positive correlations were found between MOI and the abundance of Vicinamibacterales (*r* = 0.59), Nitrososphaerales (*r* = 0.45), Rokubacteriales (*r* = 0.38) and Pyrinomonadales (*r* = 0.28), but negative correlation between MOI and the abundance of Rhizobiales (*r* = 0.61), Acidobacteriales (*r* = 0.56), Burkholderiales (*r* = 0.28) and Gaiellales (*r* = 0.22). Among fungi, Pleosporales (*r* = 0.58), Chaetothyriales (*r* = 0.39) and Microascales (*r* = 0.37) were positively correlated with MOI, while Tremellales (*r* = 0.52), Agaricales (*r* = 0.47) and Glomerellales (*r* = 0.41) displayed negative correlations.

## Discussion

In this study, we employed a tree phytometer approach by planting clonal oak saplings at two study sites in Central Germany, one of the driest regions in the country. We measured changes in plant traits, in soil physicochemical properties (in a sandy soil and a chernozem), and in soil microbial communities before and after the exceptional natural drought event in 2018.

### Soil physicochemical changes due to drought

Drought affects soil structure (e.g., soil aggregation) and chemistry (e.g., carbon content, nutrient solubility, and pH), which in turn alters the structural and functional diversity of the soil microbiome [77]. In our study, TN content increased after the drought, while NO_3_^−^-N and NH_4_^+^-N concentrations were higher before the drought. This pattern aligns with Deng et al*.*, (2021) [78], who reported that drought reduces ammonification and nitrification rates. This increase in TN may reflect reduced microbial activity responsible for nitrogen cycling, meaning that although trees took up available NO_3_^−^-N and NH_4_^+^-N, mineralization did not compensate for this uptake. Additionally, root exudates released during drought may have contributed to nitrogen and phosphorus enrichment in the soil organic matter, as noted by Chen et al. (2017) [79] and Xiong et al. (2024) [80]. Collectively, these changes suggest significant shifts in the soil microbiome as it adapts to the altered nutrient and chemical dynamics induced by drought and plant responses.

### Plant trait responses to drought

Consistent with our first hypothesis and previous studies [11–15, 43, 81–84], plant performance and most plant trait values were negatively impacted in the years following the drought. This aligns with the known drought sensitivity of *Q. robur* within European oak species [50]. Drought has been identified as the primary driver of growth decline in *Q. robur* under climate change. Research shows that growth in this species is strongly influenced by precipitation and temperature, with reduced rainfall leading to significant decreases in apical growth [85, 86]. Moreover, *Q. robur* has shown limited recovery capacity, as trees failed to return to pre-drought growth levels following spring droughts [87]. These consistent negative responses highlight the species sensitivity to water and temperature stresses and raise concerns about its long-term viability in increasingly dry climates. We also observed that better plant growth before the drought correlated with higher values of MOI and nitrogen content, suggesting that adequate MOI enhances nitrogen mobility, thereby stimulating root growth and respiration [88].

### Effects of Drought on soil microbial communities

Supporting our second hypothesis, bacterial richness and diversity were higher before the drought, regardless of tree age. This effect was more pronounced in Bad Lauchstädt’s chernozem, which has a higher water-holding capacity compared to the sandy soil of Kreinitz. This suggests that the bacterial microbiome of Bad Lauchstädt was more affected by the drought, whereas bacteria Kreinitz’ drier soil apparently exhibited pre-adapted drought resistance. Greater bacterial diversity prior to drought is generally thought to be beneficial, as increased species richness enhances metabolic functions such as nutrient mineralization and organic matter decomposition [89].

Drought also shifted bacterial community composition, favoring Gram-positive taxa, particularly Actinobacteria (Gaiellales and Solirubribacterales) and Firmicutes (Bacillales and Paenibacillales) over Gram-negative groups like Proteobacteria (Burkholderiales) and Acidobacteria (Vicinamibacterales) [18, 32, 90]. Gram-positive bacteria possess structural advantages, such as thicker cell walls and sporulation ability, enabling survival during water scarcity by entering dormant states [91, 92]. Genes involved in cell wall peptidoglycan biosynthesis increase during drought, supporting this resilience [45]. Additionally, Actinobacteria contribute to nutrient uptake, phosphate and potassium solubilization, and nitrogen fixation, further supporting plant resilience under stress [93, 94]. The order Chitinophagales (Bacteroidetes) more abundant before the drought, includes genera known as stress indicators thriving in resource-rich environments [95–98]. Although drought effects are transient and microbial communities are expected to recover [99], bacterial diversity remained depressed at least three years post-drought, confirming the severity of the 2018 drought.

Fungal diversity showed a site-specific response: richness increased during the drought period in Bad Lauchstädt, reflecting fungi’s generally better adaptation to drought compared to bacteria [38, 100]. This is based on different fungal mechanisms [41], while likewise reflecting that bacterial turnover is less resilient to drought events. Soil fungal beta diversity was influenced by tree age, but less so by drought, consistent with fungi’s closer association with plants and litter quality [101, 102]. In accordance with Bei et al*.* (2023) [45]**,** drought tended to increase the abundance of Hypocreales and Eurotiales, while the ectomycorrhizal fungal order Pezizales, known to be associated with the clonal oak DF159 [47], decreased in abundance after drought. Given that mycorrhizal fungi play a key role in nutrient and water acquisition from the soil, increasing stress tolerance [103], reduce Pezizales abundance may negatively reduce host fitness during future droughts.

### Soil properties as drivers of microbial community compositions

Distinct edaphic site characteristics are known to strongly shape soil bacterial and fungal communities [104, 105]. Our study sites differed notably in soil pH, contents of carbon, nitrogen and MOI, all determinants of microbial assemblages. Previous studies on the oak clone indicated environmental parameters have a higher impact on soil microbial communities than tree effects [47], suggesting that observed microbial differences primarily reflect environmental variation.

In our study, we identified several bacterial orders significantly correlated with physicochemical parameters: Acidobacteriales exhibited negative correlations with pH, TOC, and total TN, consistent with its known acidophilic nature and dominance in low-pH soils such as Kreinitz [106]. Vicinamibacterales positively correlated with TOC and TN, aligning with previous findings [107, 108], and were more abundant at Bad Lauchstädt, which has higher TOC and TN concentrations. Propionibacteriales, Pyrinomonadales and Rokubacteriales also correlated positively with pH and TN [110]. Rhizobiales exhibited a strong negative correlation with MOI, being more abundant after drought and particularly in Kreinitz, where MOI is lower than in Bad Lauchstädt. Rhizobiales are drought-tolerant and are often associated with nutrient-poor, low organic matter environments [109]. They can alleviate root growth inhibition caused by phosphate starvation [110].

For fungi, Tremellales and Microascales showed the strongest correlations with soil physicochemical properties and were unaffected by drought, consistent with the previous observations [111]. Tremellales, a Basidiomycota group abundant in Kreinitz alongside Agaricales, displayed negative correlations with pH and TOC, while Microascales showed opposite patterns. These site-specific distributions highlight once more the influence of environmental conditions on fungal community composition.

## Conclusion

This study highlights the profound impact of drought on soil microorganisms, plant traits and soil physicochemical properties. By using the genetically identical oak clone DF159, we were able to disentangle the effects of environmental factors such as soil type and site-specific drought conditions on microbial communities, thereby minimizing variation attributable to plant genotype.

We found that the 2018 drought significantly impacted tree growth and altered soil physicochemical properties, particularly moisture, pH, nitrogen levels, and organic carbon content, which are key drivers of microbial community composition. Notably, bacterial communities were strongly impacted by drought, exhibiting reduced diversity and significant shifts in community structure. In contrast, most fungal orders remained largely unaffected, suggesting their resilience to drought and a potential future role in supporting plant survival under water stress.

Our findings also indicate that prolonged drought stress can lead to irreversible shifts in microbial succession, with potential consequences for soil functioning and plant–microbe interactions. The results underscore the complex interplay between soil properties, microbial communities, and plant fitness in the context of climate change. This emphasizes the importance of further exploring drought-tolerant microorganisms as potential tools for enhancing plant resilience and ecosystem stability under increasing drought frequency and severity.

## Supplementary Information

Supplementary file 1.

## Data Availability

The dataset associated with the results of this article are available in the National Center for Biotechnology Information (NCBI) repository with the accession number PRJNA1205508.

## References

[1] Fierer N, Jackson RB. The diversity and biogeography of soil bacterial communities. Proc Natl Acad Sci. 2006;103:626–31. 16407148 10.1073/pnas.0507535103 PMC1334650

[2] Delgado-Baquerizo M, Maestre FT, Reich PB, Trivedi P, Osanai Y, Liu YR, et al. Carbon content and climate variability drive global soil bacterial diversity patterns. Ecol Monogr. 2016;86:373–90.

[3] Sherwood S, Fu Q. A drier future? Science. 1979;2014(343):737–9. 10.1126/science.1247620 24531959

[4] Allen CD, Macalady AK, Chenchouni H, Bachelet D, McDowell N, Vennetier M, et al. A global overview of drought and heat-induced tree mortality reveals emerging climate change risks for forests. For Ecol Manage. 2010;259:660–84.

[5] Zhao M, Running SW. Drought-induced reduction in global terrestrial net primary production from 2000 through 2009. Science. 1979;2010(329):940–3. 10.1126/science.1192666 20724633

[6] Breshears DD, Cobb NS, Rich PM, Price KP, Allen CD, Balice RG, et al. Regional vegetation die-off in response to global-change-type drought. Proc Natl Acad Sci. 2005;102:15144–8. 16217022 10.1073/pnas.0505734102 PMC1250231

[7] Van Mantgem PJ, Stephenson NL, Byrne JC, Daniels LD, Franklin JF, Fulé PZ, et al. Widespread increase of tree mortality rates in the Western United States. Science. 2009;323:521–4. 19164752 10.1126/science.1165000

[8] Bentz BJ, Rgnire J, Fettig CJ, Hansen EM, Hayes JL, Hicke JA, et al. Climate change and bark beetles of the western United States and Canada: Direct and indirect effects. Bioscience. 2010;60:602–13.

[9] Beck PSA, Juday GP, Alix C, Barber VA, Winslow SE, Sousa EE, et al. Changes in forest productivity across Alaska consistent with biome shift. Ecol Lett. 2011;14:373–9. 21332901 10.1111/j.1461-0248.2011.01598.x

[10] Schwalm CR, Williams CA, Schaefer K, Baldocchi D, Black TA, Goldstein AH, et al. Reduction in carbon uptake during turn of the century drought in western North America. Nat Geosci. 2012;5:551–6.

[11] Senf C, Buras A, Zang CS, Rammig A, Seidl R. Excess forest mortality is consistently linked to drought across Europe. Nat Commun. 2020;11:6200. 33273460 10.1038/s41467-020-19924-1 PMC7713373

[12] Obladen N, Dechering P, Skiadaresis G, Tegel W, Keßler J, Höllerl S, et al. Tree mortality of European beech and Norway spruce induced by 2018–2019 hot droughts in central Germany. Agric For Meteorol. 2021;307: 108482.

[13] Schnabel F, Purrucker S, Schmitt L, Engelmann RA, Kahl A, Richter R, et al. Cumulative growth and stress responses to the 2018–2019 drought in a European floodplain forest. Glob Chang Biol. 2022;28:1870–83. 34927360 10.1111/gcb.16028

[14] Pohl F, Werban U, Kumar R, Hildebrandt A, Rebmann C. Observational evidence of legacy effects of the 2018 drought on a mixed deciduous forest in Germany. Sci Rep. 2023;13:10863. 37407831 10.1038/s41598-023-38087-9 PMC10322863

[15] Sachsenmaier L, Schnabel F, Dietrich P, Eisenhauer N, Ferlian O, Quosh J, et al. Forest growth resistance and resilience to the 2018–2020 drought depend on tree diversity and mycorrhizal type. J Ecol. 2024;112:1787–803.

[16] Pérez Castro S, Cleland EE, Wagner R, Al SR, Lipson DA. Soil microbial responses to drought and exotic plants shift carbon metabolism. ISME J. 2019;13:1776–87. 30872806 10.1038/s41396-019-0389-9 PMC6776022

[17] Dennis PG, Miller AJ, Hirsch PR. Are root exudates more important than other sources of rhizodeposits in structuring rhizosphere bacterial communities? FEMS Microbiol Ecol. 2010;72:313–27. 20370828 10.1111/j.1574-6941.2010.00860.x

[18] Schimel J, Balser TC, Wallenstein M. Microbial stress-response physiology and its implications for ecosystem function. Ecology. 2007;88:1386–94. 17601131 10.1890/06-0219

[19] Hueso S, García C, Hernández T. Severe drought conditions modify the microbial community structure, size and activity in amended and unamended soils. Soil Biol Biochem. 2012;50:167–73.

[20] Alster CJ, German DP, Ying Lu, Allison SD. Microbial enzymatic responses to drought and to nitrogen addition in a southern California grassland. Soil Biol Biochem. 2013;64:68–79.

[21] Maestre FT, Delgado-Baquerizo M, Jeffries TC, Eldridge DJ, Ochoa V, Gozalo B, et al. Increasing aridity reduces soil microbial diversity and abundance in global drylands. PNAS. 2015;112:15684–9. 26647180 10.1073/pnas.1516684112 PMC4697385

[22] Hartmann M, Brunner I, Hagedorn F, Bardgett RD, Stierli B, Herzog C, et al. A decade of irrigation transforms the soil microbiome of a semi-arid pine forest. Mol Ecol. 2017;26:1190–206. 28028891 10.1111/mec.13995

[23] Castaño C, Lindahl BD, Alday JG, Hagenbo A, Martínez de Aragón J, Parladé J, et al. Soil microclimate changes affect soil fungal communities in a Mediterranean pine forest. New Phytol. 2018;220:1211–21. 29757469 10.1111/nph.15205

[24] Stark JM, Firestone MK. Mechanisms for soil moisture effects on activity of nitrifying bacteria. Appl Environ Microbiol. 1995;61:218–21. 16534906 10.1128/aem.61.1.218-221.1995 PMC1388328

[25] Sardans J, Peñuelas J. Increasing drought decreases phosphorus availability in an evergreen Mediterranean forest. Plant Soil. 2004;267:367–77.

[26] Ford DJ, Cookson WR, Adams MA, Grierson PF. Role of soil drying in nitrogen mineralization and microbial community function in semi-arid grasslands of north-west Australia. Soil Biol Biochem. 2007;39:1557–69.

[27] Van der Molen MK, Dolman AJ, Ciais P, Eglin T, Gobron N, Law BE, et al. Drought and ecosystem carbon cycling. Agric For Meteorol. 2011;151:765–73.

[28] Sardans J, Peñuuelas J, Estiarte M, Prieto P. Warming and drought alter C and N concentration, allocation and accumulation in a Mediterranean shrubland. Glob Chang Biol. 2008;14:2304–16.

[29] Fuchslueger L, Bahn M, Fritz K, Hasibeder R, Richter A. Experimental drought reduces the transfer of recently fixed plant carbon to soil microbes and alters the bacterial community composition in a mountain meadow. New Phytol. 2014;201:916–27. 24171922 10.1111/nph.12569 PMC3908363

[30] Hayden HL, Mele PM, Bougoure DS, Allan CY, Norng S, Piceno YM, et al. Changes in the microbial community structure of bacteria, archaea and fungi in response to elevated CO2 and warming in an Australian native grassland soil. Environ Microbiol. 2012;14:3081–96. 23039205 10.1111/j.1462-2920.2012.02855.x

[31] Marasco R, Rolli E, Ettoumi B, Vigani G, Mapelli F, Borin S, et al. A Drought resistance-promoting microbiome is selected by root system under desert farming. PLoS ONE. 2012;7: e48479. 23119032 10.1371/journal.pone.0048479 PMC3485337

[32] Acosta-Martínez V, Cotton J, Gardner T, Moore-Kucera J, Zak J, Wester D, et al. Predominant bacterial and fungal assemblages in agricultural soils during a record drought/heat wave and linkages to enzyme activities of biogeochemical cycling. Appl Soil Ecol. 2014;84:69–82.

[33] Jones PD, Lister DH, Jaggard KW, Pidgeon JD. Future climate impact on the productivity of sugar beet (Beta vulgaris L.) in Europe. Clim Change. 2003;58:93–108.

[34] Moretti CL, Mattos LM, Calbo AG, Sargent SA. Climate changes and potential impacts on postharvest quality of fruit and vegetable crops: A review. Food Res Int. 2010;43:1824–32.

[35] Gauvry E, Mathot AG, Leguérinel I, Couvert O, Postollec F, Broussolle V, et al. Knowledge of the physiology of spore-forming bacteria can explain the origin of spores in the food environment. Res Microbiol. 2017;168:369–78. 27810476 10.1016/j.resmic.2016.10.006

[36] Yuste JC, Peñuelas J, Estiarte M, Garcia-Mas J, Mattana S, Ogaya R, et al. Drought-resistant fungi control soil organic matter decomposition and its response to temperature. Glob Chang Biol. 2011;17:1475–86.

[37] Barnard RL, Osborne CA, Firestone MK. Changing precipitation pattern alters soil microbial community response to wet-up under a Mediterranean-type climate. ISME J. 2015;9:946–57. 25314319 10.1038/ismej.2014.192 PMC4817701

[38] Treseder KK, Berlemont R, Allison SD, Martiny AC. Drought increases the frequencies of fungal functional genes related to carbon and nitrogen acquisition. PLoS ONE. 2018;13: e0206441. 30462680 10.1371/journal.pone.0206441 PMC6248904

[39] de Vries FT, Griffiths RI, Bailey M, Craig H, Girlanda M, Gweon HS, et al. Soil bacterial networks are less stable under drought than fungal networks. Nat Commun. 2018;9:3033. 30072764 10.1038/s41467-018-05516-7 PMC6072794

[40] Barnard RL, Osborne CA, Firestone MK. Responses of soil bacterial and fungal communities to extreme desiccation and rewetting. ISME J. 2013;7:2229–41. 23823489 10.1038/ismej.2013.104 PMC3806258

[41] Treseder KK, Lennon JT. Fungal traits that drive ecosystem dynamics on land. Microbiol Mol Biol Rev. 2015;79:243–62. 25971588 10.1128/MMBR.00001-15 PMC4429240

[42] Begum N, Qin C, Ahanger MA, Raza S, Khan MI, Ashraf M, et al. Role of arbuscular mycorrhizal fungi in plant growth regulation: implications in abiotic stress tolerance. Front Plant Sci. 2019;10:1068. 31608075 10.3389/fpls.2019.01068 PMC6761482

[43] Schuldt B, Buras A, Arend M, Vitasse Y, Beierkuhnlein C, Damm A, et al. A first assessment of the impact of the extreme 2018 summer drought on Central European forests. Basic Appl Ecol. 2020;45:86–103.

[44] Süßel F, Brüggemann W. Tree water relations of mature oaks in southwest Germany under extreme drought stress in summer 2018. Plant Stress. 2021;1: 100010.

[45] Bei Q, Reitz T, Schnabel B, Eisenhauer N, Schädler M, Buscot F, et al. Extreme summers impact cropland and grassland soil microbiomes. ISME J. 2023;17:1589–600. 37419993 10.1038/s41396-023-01470-5 PMC10504347

[46] Herrmann S, Grams TEE, Tarkka MT, Angay O, Bacht M, Bönn M, et al. Endogenous rhythmic growth, a trait suitable for the study of interplays between multitrophic interactions and tree development. Perspect Plant Ecol Evol Syst. 2016;19:40–8.

[47] de Habiyaremye JD, Goldmann K, Reitz T, Herrmann S, Buscot F. Tree root zone microbiome: exploring the magnitude of environmental conditions and host tree impact. Front Microbiol. 2020;11:749. 32390986 10.3389/fmicb.2020.00749 PMC7190799

[48] Hamer U, Makeschin F, Stadler J, Klotz S. Soil organic matter and microbial community structure in set-aside and intensively managed arable soils in NE-Saxony. Germany Appl Soil Ecol. 2008;40:465–75.

[49] Altermann M, Rinklebe J, Merbach I, Körschens M, Langer U, Hofmann B. Chernozem - soil of the year 2005. J Plant Nutr Soil Sci. 2005;168:725–40.

[50] Konatowska M, Młynarczyk A, Rutkowski P, Kujawa K. Impact of site conditions on quercus robur and quercus petraea growth and distribution under global climate change. Remote Sens (Basel). 2024;16:4094.

[51] Herrmann S, Munch JC, Buscot F. A gnotobiotic culture system with oak microcuttings to study specific effects of mycobionts on plant morphology before, and in the early phase of, ectomycorrhiza formation Paxillus involutus and Piloderma croceum. New Phytol. 1998;138:203–12. 33863094 10.1046/j.1469-8137.1998.00105.x

[52] de Habiyaremye JD, Herrmann S, Reitz T, Buscot F, Goldmann K. Balance between geographic, soil, and host tree parameters to shape soil microbiomes associated to clonal oak varies across soil zones along a European North-South transect. Environ Microbiol. 2021;23:2274–92. 33587815 10.1111/1462-2920.15433

[53] Ghani A, Dexter M, Perrott KW. Hot-water extractable carbon in soils: A sensitive measurement for determining impacts of fertilisation, grazing and cultivation. Soil Biol Biochem. 2003;35:1231–43.

[54] Schulz E, Breulmann M, Boettger T, Wang KR, Neue HU. Effect of organic matter input on functional pools of soil organic carbon in a long-term double rice crop experiment in China. Eur J Soil Sci. 2011;62:134–43.

[55] Zsolnay A. Dissolved humus in soil waters. In: Humic Substances in Terrestrial Ecosystems. Elsevier; 1996. pp 171–223.

[56] Zakharova A, Beare MH, Cieraad E, Curtin D, Turnbull MH, Millard P. Factors controlling labile soil organic matter vulnerability to loss following disturbance as assessed by measurement of soil-respired δ13CO2. Eur J Soil Sci. 2015;66:135–44.

[57] Schmidt J, Fester T, Schulz E, Michalzik B, Buscot F, Gutknecht J. Effects of plant-symbiotic relationships on the living soil microbial community and microbial necromass in a long-term agro-ecosystem. Sci Total Environ. 2017;581–582:756–65. 28082056 10.1016/j.scitotenv.2017.01.005

[58] Caporaso JG, Lauber CL, Walters WA, Berg-Lyons D, Huntley J, Fierer N, et al. Ultra-high-throughput microbial community analysis on the Illumina HiSeq and MiSeq platforms. ISME J. 2012;6:1621–4. 22402401 10.1038/ismej.2012.8 PMC3400413

[59] Moll J, Kellner H, Leonhardt S, Stengel E, Dahl A, Bässler C, et al. Bacteria inhabiting deadwood of 13 tree species are heterogeneously distributed between sapwood and heartwood. Environ Microbiol. 2018;20:3744–56. 30109768 10.1111/1462-2920.14376

[60] Gardes M, Bruns TD. ITS primers with enhanced specificity for basidiomycetes - application to the identification of mycorrhizae and rusts. Mol Ecol. 1993;2:113–8. 8180733 10.1111/j.1365-294x.1993.tb00005.x

[61] Ihrmark K, Bödeker ITM, Cruz-Martinez K, Friberg H, Kubartova A, Schenck J, et al. New primers to amplify the fungal ITS2 region - evaluation by 454-sequencing of artificial and natural communities. FEMS Microbiol Ecol. 2012;82:666–77. 22738186 10.1111/j.1574-6941.2012.01437.x

[62] Leonhardt S, Hoppe B, Stengel E, Noll L, Moll J, Bässler C, et al. Molecular fungal community and its decomposition activity in sapwood and heartwood of 13 temperate European tree species. PLoS ONE. 2019;14: e0212120. 30763365 10.1371/journal.pone.0212120 PMC6375594

[63] Weißbecker C, Schnabel B, Heintz-Buschart A. Dadasnake a snakemake implementation of DADA2 to process amplicon sequencing data for microbial ecology. Gigascience. 2020;9:giaa135. 33252655 10.1093/gigascience/giaa135 PMC7702218

[64] Callahan BJ, McMurdie PJ, Rosen MJ, Han AW, Johnson AJA, Holmes SP. DADA2: High-resolution sample inference from Illumina amplicon data. Nat Methods. 2016;13:581–3. 27214047 10.1038/nmeth.3869 PMC4927377

[65] Estensmo ELF, Maurice S, Morgado L, Martin-Sanchez PM, Skrede I, Kauserud H. The influence of intraspecific sequence variation during DNA metabarcoding: A case study of eleven fungal species. Mol Ecol Resour. 2021;21:1141–8. 33459491 10.1111/1755-0998.13329

[66] Schloss PD. Amplicon sequence variants artificially split bacterial genomes into separate clusters. MSphere. 2021;6:10–1128. 10.1128/mSphere.00191-21 PMC8386465 34287003

[67] Quast C, Pruesse E, Yilmaz P, Gerken J, Schweer T, Yarza P, et al. The SILVA ribosomal RNA gene database project: Improved data processing and web-based tools. Nucleic Acids Res. 2013;41:D590–6. 23193283 10.1093/nar/gks1219 PMC3531112

[68] Nilsson RH, Larsson KH, Taylor AFS, Bengtsson-Palme J, Jeppesen TS, Schigel D, et al. The UNITE database for molecular identification of fungi: Handling dark taxa and parallel taxonomic classifications. Nucleic Acids Res. 2019;47:D259–64. 30371820 10.1093/nar/gky1022 PMC6324048

[69] R Core Team. R: A language and environment for statistical computing. 2023.

[70] Bates D, Mächler M, Bolker BM, Walker SC. Fitting linear mixed-effects models using lme4. J Stat Softw. 2015;67:1–48.

[71] Kim S. ppcor: An R package for a fast calculation to semi-partial correlation coefficients. Commun Stat Appl Methods. 2015;22:665–74. 26688802 10.5351/CSAM.2015.22.6.665 PMC4681537

[72] McMurdie PJ, Holmes S. Phyloseq: An R package for reproducible interactive analysis and graphics of microbiome census data. PLoS ONE. 2013;8: e61217. 23630581 10.1371/journal.pone.0061217 PMC3632530

[73] Hill MO. Diversity and evenness: a unifying notation and its consequences. Ecology. 1973;54:427–32.

[74] Alberdi A, Gilbert MTP. hilldiv: an R package for the integral analysis of diversity based on Hill numbers. Biorxiv. 2019; 545665.

[75] Kindt R, Coe R. Tree diversity analysis: a manual and software for common statistical methods for ecological and biodiversity studies. World Agroforestry Centre; 2005.

[76] Segata N, Izard J, Waldron L, Gevers D, Miropolsky L, Garrett WS, et al. Metagenomic biomarker discovery and explanation. Genome Biol. 2011;12:1–18. 10.1186/gb-2011-12-6-r60 PMC3218848 21702898

[77] Bogati K, Walczak M. The impact of drought stress on soil microbial community. Enzyme Activit Plants Agronomy. 2022;12:189.

[78] Deng L, Peng C, Kim DG, Li J, Liu Y, Hai X, et al. Drought effects on soil carbon and nitrogen dynamics in global natural ecosystems. Earth Sci Rev. 2021;214: 103501.

[79] Chen J, Xiao G, Kuzyakov Y, Jenerette D, Ma Y, Liu W, et al. Soil nitrogen transformation responses to seasonal precipitation changes are regulated by changes in functional microbial abundance in a subtropical forest. Biogeosciences. 2017;14:2513–25.

[80] Xiong Y, Zhang Y, Zhang Z, Feng T, Wang P, Keesstra S. The effects and contributions of ecological factors on soil carbon, water and nutrient storages under long-term vegetation restoration on the eastern loess plateau. Forests. 2024;15:1898.

[81] Gordon DR, Welker JM, Menke JW, Rice KJ. Competition for soil water between annual plants and blue oak ( Quercus douglasix) seedlings. Oecologia. 1989;79:533–41. 28313489 10.1007/BF00378672

[82] Dickson RE, Tomlinson PT. Oak growth, development and carbon metabolism in response to water stress. 1996;53:181–96

[83] Spieß N, Oufir M, Matušíková I, Stierschneider M, Kopecky D, Homolka A, et al. Ecophysiological and transcriptomic responses of oak (Quercus robur) to long-term drought exposure and rewatering. Environ Exp Bot. 2012;77:117–26.

[84] Buras A, RammigZang ASC. Quantifying impacts of the 2018 drought on European ecosystems in comparison to 2003. Biogeosciences. 2020;2020(17):1655–72.

[85] Arend M, Kuster T, Günthardt-Goerg MS, Dobbertin M. Provenance-specific growth responses to drought and air warming in three European oak species (Quercus robur, Q. petraea and Q. pubescens). Tree Physiol. 2011;31:287–97. 21422189 10.1093/treephys/tpr004

[86] Scharnweber T, Manthey M, Criegee C, Bauwe A, Schröder C, Wilmking M. Drought matters - declining precipitation influences growth of Fagus sylvatica L. and Quercus robur L. in north-eastern Germany. For Ecol Manage. 2011;262:947–61.

[87] Bose AK, Scherrer D, Camarero JJ, Ziche D, Babst F, Bigler C, et al. Climate sensitivity and drought seasonality determine post-drought growth recovery of Quercus petraea and Quercus robur in Europe. Sci Total Environ. 2021;784:147222. 34088042 10.1016/j.scitotenv.2021.147222

[88] Valliere JM, Allen EB. Interactive effects of nitrogen deposition and drought-stress on plant-soil feedbacks of Artemisia californica seedlings. Plant Soil. 2016;403:277–90.

[89] Zhang H, Jiang N, Zhang S, Zhu X, Wang H, Xiu W, et al. Soil bacterial community composition is altered more by soil nutrient availability than pH following long-term nutrient addition in a temperate steppe. Front Microbiol. 2024;15:1455891. 39345260 10.3389/fmicb.2024.1455891 PMC11427344

[90] Chodak M, Gołębiewski M, Morawska-Płoskonka J, Kuduk K, Niklińska M. Soil chemical properties affect the reaction of forest soil bacteria to drought and rewetting stress. Ann Microbiol. 2015;65:1627–37. 26273241 10.1007/s13213-014-1002-0 PMC4529456

[91] Potts M. Desiccation tolerance of prokaryotes. Microbiol Rev. 1994;58:755–805. 7854254 10.1128/mr.58.4.755-805.1994 PMC372989

[92] Naylor D, Coleman-Derr D. Drought stress and root-associated bacterial communities. Front Plant Sci. 2018;8:2223. 29375600 10.3389/fpls.2017.02223 PMC5767233

[93] Etesami H, Adl SM. Plant Growth-Promoting Rhizobacteria (PGPR) and Their Action Mechanisms in Availability of Nutrients to Plants. In: Kumar M, Kumar V, Prasad R, editors. Phyto-Microbiome in Stress Regulation. Singapore: Springer; 2020. p. 147–203.

[94] Boubekri K, Soumare A, Mardad I, Lyamlouli K, Ouhdouch Y, Hafidi M, et al. Multifunctional role of Actinobacteria in agricultural production sustainability: A review. Microbiol Res. 2022;261: 127059. 35584559 10.1016/j.micres.2022.127059

[95] Duchow E, Douglas HC. Rhodomicrobium vannielii, a new photoheterotrophic bacterium. J Bacteriol. 1949;58:409–16. 16561801 10.1128/jb.58.4.409-416.1949 PMC385647

[96] Qu JH, Yuan HL. Sediminibacterium salmoneum gen. nov., sp. Nov., a member of the phylum Bacteroidetes isolated from sediment of a eutrophic reservoir. Int J Syst Evol Microbiol. 2008;58:2191–4. 18768628 10.1099/ijs.0.65514-0

[97] Kim SJ, Moon JY, Lim JM, Hamada M, Ahn JH, Weon HY, et al. Jatrophihabitans soli sp. nov., isolated from soil. Int J Syst Evol Microbiol. 2015;65:1759–63. 25744581 10.1099/ijs.0.000173

[98] Papp K, Hungate BA, Schwartz E. Glucose triggers strong taxon-specific responses in microbial growth and activity: insights from DNA and RNA qSIP. Ecology. 2020;101: e02887. 31502670 10.1002/ecy.2887

[99] Shade A, Peter H, Allison SD, Baho DL, Berga M, Bürgmann H, et al. Fundamentals of microbial community resistance and resilience. Front Microbiol. 2012;3:417. 23267351 10.3389/fmicb.2012.00417 PMC3525951

[100] Wu Z, Dijkstra P, Koch GW, Peñuelas J, Hungate BA. Responses of terrestrial ecosystems to temperature and precipitation change: A meta-analysis of experimental manipulation. Glob Chang Biol. 2011;17:927–42.

[101] Sun S, Li S, Avera BN, Strahm BD, Badgley BD. Soil bacterial and fungal communities show distinct recovery patterns during forest ecosystem restoration. Appl Environ Microbiol. 2017;83:e00966-e1017. 28476769 10.1128/AEM.00966-17 PMC5494632

[102] Hu Y, Zhang X, Chen H, Jiang Y, Zhang J. Effects of forest age and season on soil microbial communities in Chinese fir plantations. Microbiol Spectr. 2024;12:e04075-e4123. 38980023 10.1128/spectrum.04075-23 PMC11302042

[103] Tedersoo L, May TW, Smith ME. Ectomycorrhizal lifestyle in fungi: global diversity, distribution, and evolution of phylogenetic lineages. Mycorrhiza. 2010;20:217–63. 20191371 10.1007/s00572-009-0274-x

[104] Bokulich NA, Thorngate JH, Richardson PM, Mills DA. Microbial biogeography of wine grapes is conditioned by cultivar, vintage, and climate. Proc Natl Acad Sci. 2014;111:E139–48. 24277822 10.1073/pnas.1317377110 PMC3890796

[105] Gourmelon V, Maggia L, Powell JR, Gigante S, Hortal S, Gueunier C, et al. Environmental and geographical factors structure soil microbial diversity in new caledonian ultramafic substrates: A metagenomic approach. PLoS ONE. 2016;11: e0167405. 27907121 10.1371/journal.pone.0167405 PMC5131939

[106] Kalam S, Basu A, Ahmad I, Sayyed RZ, El-Enshasy HA, Dailin DJ, et al. Recent understanding of soil acidobacteria and their ecological significance: a critical review. Front Microbiol. 2020;11: 100173. 10.3389/fmicb.2020.580024 PMC7661733 33193209

[107] Hu S, Song G, Gao J, Wang Y, Yang Q, Qiu R, et al. Characteristics of DOM and bacterial community in rural black and odorous water bodies under different dimensions. Sci Total Environ. 2024;927:172005. 38554969 10.1016/j.scitotenv.2024.172005

[108] Niu H, Yuan M, Chen X, Zhao J, Cui Y, Song Y, et al. Deciphering the differences of bacterial communities between high- and low-productive wheat fields using high-throughput sequencing. Front Microbiol. 2024;15:1391428. 39296300 10.3389/fmicb.2024.1391428 PMC11408337

[109] Zhao C, He X, Dan X, He M, Zhao J, Meng H, et al. Soil dissolved organic matters mediate bacterial taxa to enhance nitrification rates under wheat cultivation. Sci Total Environ. 2022;828:3967446. 10.1016/j.scitotenv.2022.154418 35276137

[110] Garrido-Oter R, Nakano RT, Dombrowski N, Ma KW, McHardy AC, Schulze-Lefert P. Modular traits of the rhizobiales root microbiota and their evolutionary relationship with symbiotic rhizobia. Cell Host Microbe. 2018;24:155-167.e5. 30001518 10.1016/j.chom.2018.06.006 PMC6053594

[111] Louw N, Gherardi LA, Sala OE, Chung YA. Dryland soil mycobiome response to long-term precipitation variability depends on host type. J Ecol. 2022;110:2984–97.

