# Supplementary material for: Severe drought impacts tree traits and associated soil microbial communities of clonal oaks
Source: Environ Microbiome. 2025 Jun 6;20:63. doi: 10.1186/s40793-025-00720-7 (PMC12143084; doi:10.1186/s40793-025-00720-7)
Supplement: Supplementary file 1 [file 40793_2025_720_MOESM1_ESM.docx]

**Severe drought impact traits and associated soil microbial communities of clonal oaks**

Camilo Quiroga-González^1§^, Luis Daniel Prada-Salcedo^1^, François Buscot^1,2^, Mika Tarkka^1,2^, Sylvie Herrmann^1^, Marie-Lara Bouffaud^1*^ and Kezia Goldmann^1*^

**Supplemental material**

Table S1. Sample overview: The columns provide information of the drought period (before and after the drought in 2018), and the plot where the samples were collected; P1 = Plot1 and P2 = Plot2. The rows have the information of the study site and the year of the trees after outplanting.

|  | **Before drought** | | | **After drought** | | | **Total number of samples** |
| --- | --- | --- | --- | --- | --- | --- | --- |
|  | **P1** | **P2** | **Total** | **P1** | **P2** | **Total** |  |
| **Bad Lauchstädt** | 46 | 12 | 58 | 6 | 48 | 54 | **112** |
| 1 year | 4 | 6 | 10 |  | 6 | 6 | 16 |
| 2 year | 6 | 6 | 12 |  | 12 | 12 | 24 |
| 3 year | 12 |  | 12 |  | 12 | 12 | 24 |
| 4 year | 12 |  | 12 |  | 12 | 12 | 24 |
| 5 year | 12 |  | 12 | 6 | 6 | 12 | 24 |
|  |  |  |  |  |  |  |  |
| **Kreinitz** | 49 | 12 | 61 | 6 | 47 | 53 | **114** |
| 1 year | 6 | 6 | 12 |  | 6 | 6 | 18 |
| 2 year | 6 | 6 | 12 |  | 12 | 12 | 24 |
| 3 year | 12 |  | 12 |  | 12 | 12 | 24 |
| 4 year | 12 |  | 12 |  | 11 | 11 | 23 |
| 5 year | 13 |  | 13 | 6 | 6 | 12 | 25 |
|  |  |  |  |  |  |  |  |
| **Both sites combined** | 95 | 24 | **119** | 12 | 95 | **107** | **226** |

Table S2. Comparison of soil chemical variables between sampling sites. Bold results show significantly higher value (linear mix model, *P*<0.05) in that specific site.

|  | **Bad Lauchstädt** | | **Kreinitz** | |
| --- | --- | --- | --- | --- |
| **Variable** | Mean | Std. Error | Mean | Std. Error |
| pH | **6.598** | **0.484** | 5.130 | 0.276 |
| Soil Moisture (MOI) | **11.817** | **3.563** | 5.395 | 3.059 |
| Soil Dry Matter (DMC) | 97.783 | 0.461 | **99.376** | **0.147** |
| Ammonium Nitrogen Content (NH_4_^+^-N) | 1.157 | 0.783 | **1.506** | **1.332** |
| Nitrate Nitrogen Content (NO_3_^-^−N) | **3.263** | **3.889** | 2.774 | 4.051 |
| Inorganic Nitrogen Content (N_min_) | 4.420 | 3.848 | 4.280 | 4.853 |
| Hot Water Extractable Organic Carbon (HWC) | 427.062 | 76.028 | **449.671** | **108.235** |
| Hot Water Extractable Nitrogen (HWN) | 41.746 | 13.788 | 39.342 | 11.089 |
| HWC/HWN | 10.912 | 2.4606 | **11.659** | **2.047** |
| Cold Water Extractable Organic Carbon (CWC) | 54.429 | 18.280 | 53.503 | 17.026 |
| Cold Water Extractable Nitrogen (CWN) | 6.570 | 3.180 | 7.315 | 3.859 |
| CWC/CWN | **9.634** | **4.0406** | 8.476 | 3.152 |
| Total Soil Organic Carbon (TOC) | **1.866** | **0.191** | 0.847 | 0.159 |
| Total Soil Nitrogen (TN) | **0.135** | **0.0186** | 0.0719 | 0.019 |
| TOC/TN | **13.968** | **1.645** | 12.338 | 2.852 |

Table S3. Comparison of each soil chemical variable between the two drought periods “Before” and “After”. Bold results show a significantly higher value (linear mix model, *P*<0.05).

|  | **Before drought** | | **After drought** | |
| --- | --- | --- | --- | --- |
| **Variables** | Mean | Std. Error | Mean | Std. Error |
| pH | 6.062 | 0.900 | 5.592 | 0.690 |
| Soil Moisture (MOI) | **9.793** | **4.643** | 7.135 | 4.194 |
| Soil Dry Matter (DMC) | 98.613 | 0.871 | 98.658 | 0.854 |
| Ammonium Nitrogen Content (NH_4_^+^-N) | **1.829** | **1.099** | 0.921 | 0.964 |
| Nitrate Nitrogen Content (NO_3_^-^−N) | **3.386** | **4.492** | 2.664 | 3.447 |
| Inorganic Nitrogen Content (N_min_) | **5.215** | **4.987** | 3.585 | 3.684 |
| Hot Water Extractable Organic Carbon (HWC) | 420.144 | 97.705 | 455.803 | 89.987 |
| Hot Water Extractable Nitrogen (HWN) | 43.796 | 13.995 | 37.543 | 10.104 |
| HWC/HWN | 9.926 | 1.498 | 12.524 | 2.147 |
| Cold Water Extractable Organic Carbon (CWC) | 48.079 | 16.597 | **60.168** | **16.506** |
| Cold Water Extractable Nitrogen (CWN) | 6.753 | 3.895 | 7.198 | 3.193 |
| CWC/CWN | 8.453 | 3.310 | **9.612** | **3.869** |
| Total Soil Organic Carbon (TOC) | 1.289 | 0.557 | 1.347 | 0.521 |
| Total Soil Nitrogen (TN) | 0.090 | 0.039 | **0.111** | **0.0314** |
| TOC/TN | **14.499** | **2.336** | 11.867 | 1.939 |

Table S4. Comparison between plant traits and sampling location. Bold results show a significantly higher value (linear mix model, *P*<0.05).

|  | **Bad Lauchstädt** | | **Kreinitz** | |
| --- | --- | --- | --- | --- |
| **Variable** | Mean | Std. Error | Mean | Std. Error |
| Leaf Area | 18.053 | 7.662 | 18.130 | 6.264 |
| Number of Leaves/SF | **4.9802** | **0.173** | 4.893 | 0.540 |
| Leaf Dry Weight | 1.105 | 0.348 | 1.029 | 0.422 |
| Number of Shoot Flushes | **2.162** | **0.842** | 1.982 | 0.771 |
| Branch Elongation | 32.962 | 22.545 | 32.910 | 21.789 |
| Apical Growth | 71.069 | 49.471 | 63.330 | 46.199 |
| Shoot Flush Length | 28.162 | 14.411 | 28.340 | 13.568 |

Table S5. Comparison between plant traits and drought period. Bold results show a significantly higher value (linear mix model, *P*<0.05).

|  | **Before drought** | | **After drought** | |
| --- | --- | --- | --- | --- |
| **Variable** | Mean | Std. Error | Mean | Std. Error |
| Leaf Area | 17.506 | 6.953 | **18.755** | **6.830** |
| Number of Leaves | **4.976** | **0.155** | 4.883 | 0.585 |
| Leaves Dry Weight | **1.112** | **0.357** | 1.009 | 0.421 |
| Number of Shoot Flush | **2.365** | **0.765** | 1.728 | 0.718 |
| Branch Elongation | **40.268** | **21.343** | 27.616 | 20.980 |
| Apical Growth | **81.677** | **46.280** | 59.919 | 47.225 |
| Length Shoot Flush | **31.894** | **13.202** | 19.301 | 11.336 |


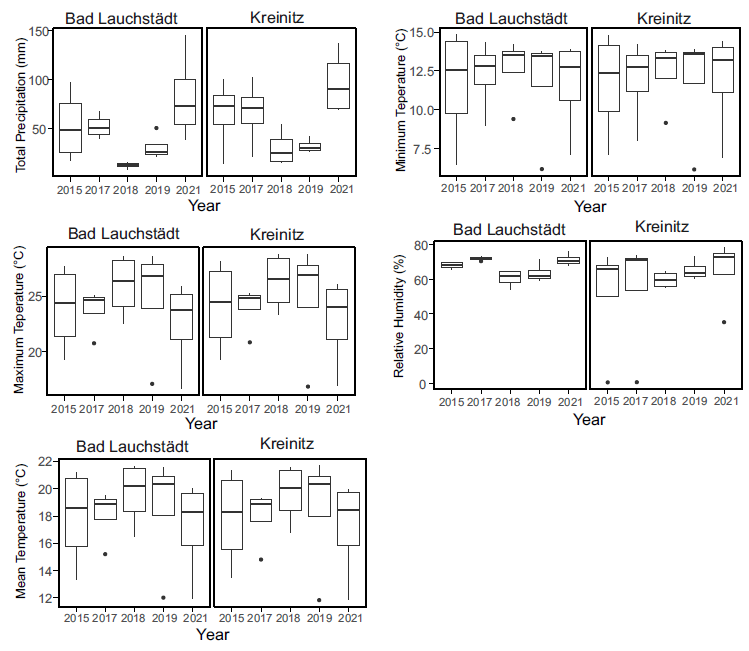


*Figure S2. Differences in environmental variables in between years for each sampling site. The values in °C and % represent the mean while the other variables mm represent the cumulative value. Yearly values are filtered from the month of May to August*

Figure S1. Differences in environmental variables in between years for each sampling site. Values for temperature and humidity represent means, while the precipitation is represented as cumulative values. Annual values are filtered from the months May to August.


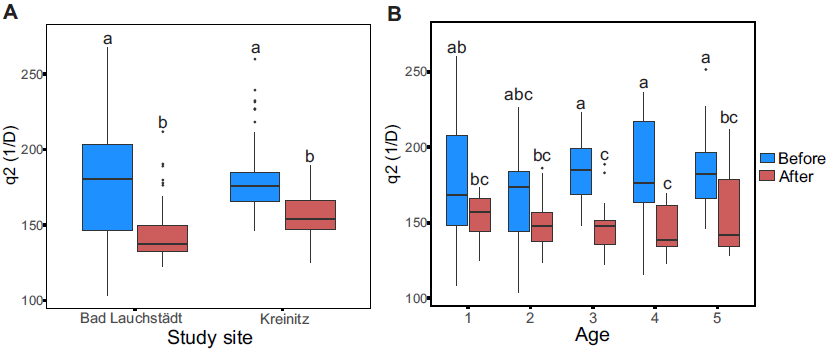
Figure S2. Boxplot showing the distribution of inverse Simpson index of bacteria A) study site and B) age. The boxes represent the quartiles (25%, 50% and 75%), and the whiskers indicate the range of the data, excluding outliers. Blue color represents the values before the drought and the red color after. Letters above boxplots represent significant differences according to ANOVA analysis.

*Figure S3. Boxplot showing the distribution of q0 in A) Bacteria and B) Fungi for each location site. The boxes represent the quartiles (25%, 50% and 75%), and the whiskers indicate the range of the data, excluding outliers. Blue color represents the values before the drought and the red color after.*

*Figure S4. Inverse of Simpson Index for Bacteria A) diversity of OTU in relation to study site and B) diversity of OTU in relation to oak age. The colors represent the period of the collected sample.*


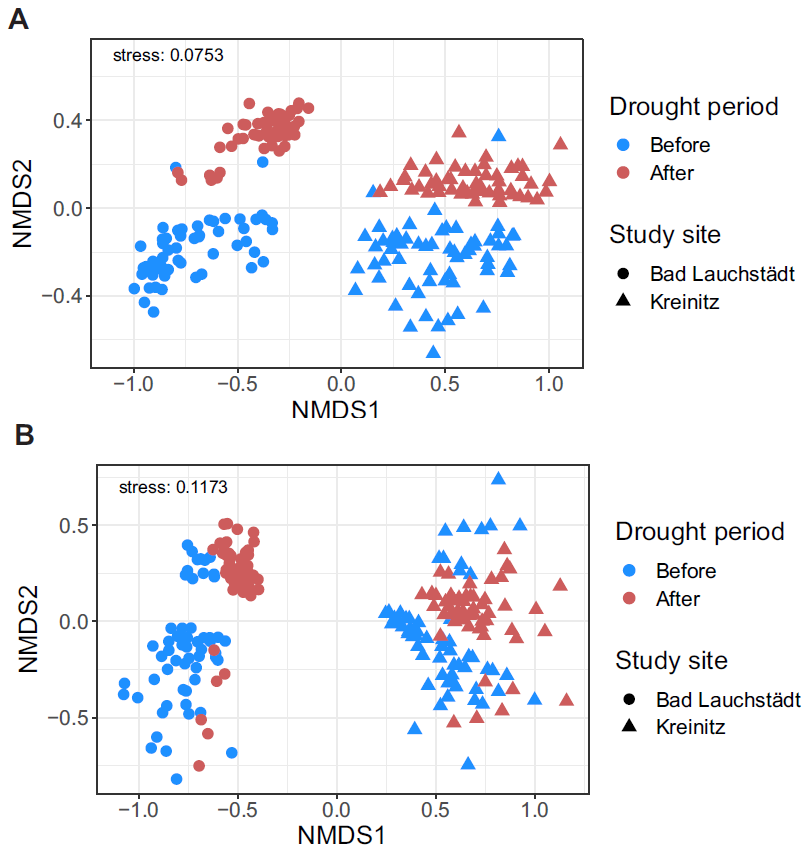


Figure S3. NMDS of the microbial communities based on OTU from A) bacteria and B) fungi. The symbols represent the study site and the colors the drought period.

*Figure S5. NMDS of the microbial communities based on OTU from A) Bacteria and B) Fungi. The forms represent the study site and the colors the drought period.*

Figure S4. NMDS of the microbial communities based on OTU abundance from A) bacteria and B) fungi. The symbols represent the study site and the colors the year of sampling.


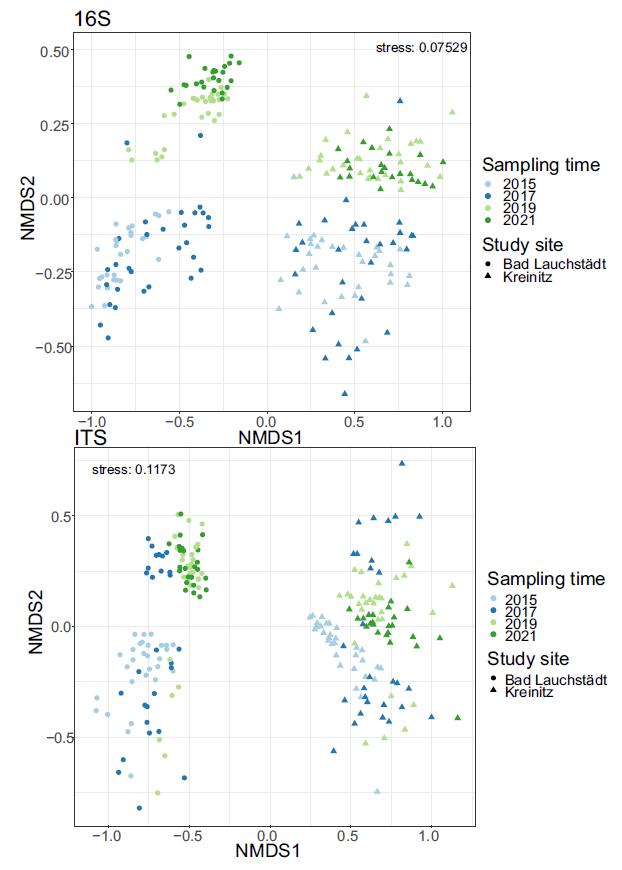


A

B

*Figure S6. NMDS of the microbial communities based on OUT from A) Bacteria and B) Fungi. The forms represent the study site and the colors the year of sampling.*
